# Frontiers in biomolecular mesh generation and molecular visualization systems

**DOI:** 10.1186/s42492-018-0007-0

**Published:** 2018-09-05

**Authors:** Sheng Gui, Dawar Khan, Qin Wang, Dong-Ming Yan, Ben-Zhuo Lu

**Affiliations:** 1 LSEC, NCMIS, Academy of Mathematics and Systems Science, Chinese Academy of Sciences, Beijing, 100190 China; 2 National Laboratory of Pattern Recognition, Institute of Automation, Chinese Academy of Sciences, Beijing, 100190 China; 3 University of Chinese Academy of Sciences, Beijing, 100049 China

**Keywords:** Biomolecular mesh generation, Remeshing, BEM, FEM, Molecular visualization

## Abstract

With the development of biomolecular modeling and simulation, especially implicit solvent modeling, higher requirements are set for the stability, efficiency and mesh quality of molecular mesh generation software. In this review, we summarize the recent works in biomolecular mesh generation and molecular visualization. First, we introduce various definitions of molecular surface and corresponding meshing software. Second, as the mesh quality significantly influences biomolecular simulation, we investigate some remeshing methods in the fields of computer graphics and molecular modeling. Then, we show the application of biomolecular mesh in the boundary element method (BEM) and the finite element method (FEM). Finally, to conveniently visualize the numerical results based on the mesh, we present two types of molecular visualization systems.

## Background

With the development of biophysics and computational sciences, numerical simulation methods are widely applied in the biomolecular field, such as the transport processes within ion channels and the electrostatic interactions of proteins. The typically used methods include molecular dynamics [1–3], Brownian motion simulation [4–7] and continuous models [8–11]. Debye and Hückel [12] proposed a continuous model in 1923. In this model, ions are considered to be continuously distributed, and two types of equations including the Poisson-Boltzmann (PB) equation and the Poisson-Nernst-Planck (PNP) [8] equations are deduced. By solving these two equations, the electrostatic interactions of molecules and the diffusion processes of ions can be simulated.

**Fig. 1:**
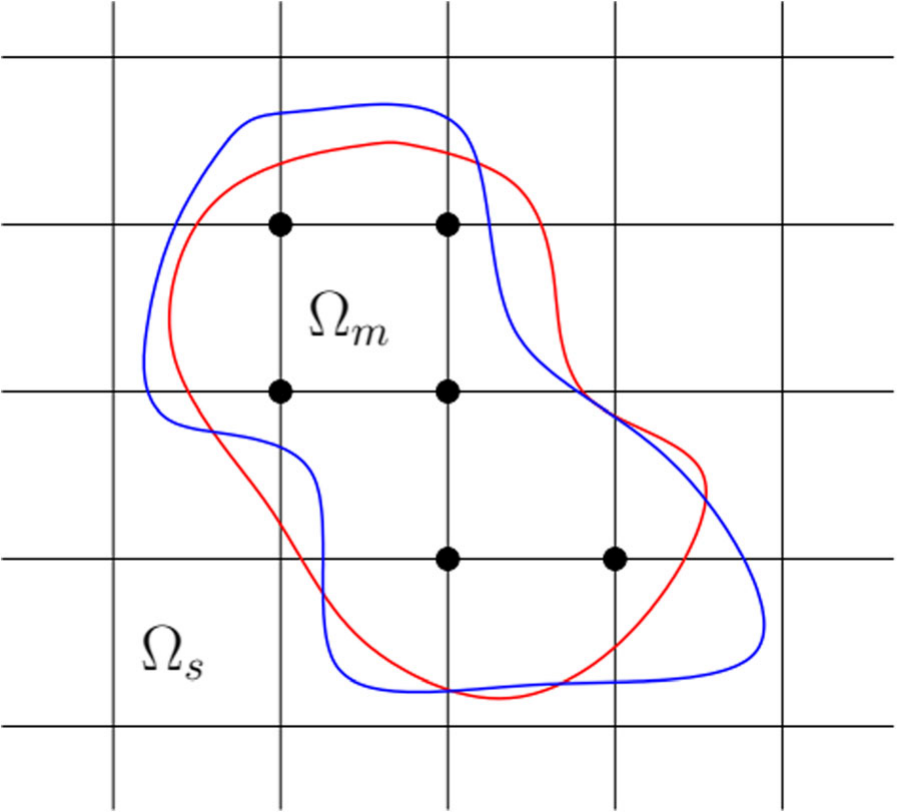
The resolution of molecular surface in the traditional finite difference method for the PB equation is limited by the mesh size. Two completely different molecular surfaces could give identical maps of dielectric constants on the same mesh. The grid nodes in the solute are marked with black dots

A number of numerical methods have been used to solve the continuous model, including the finite element method (FEM) [8, 13–16], finite difference method (FDM) [17, 18] and boundary element method (BEM) [19–21]. In comparison with other methods, the FEM is simpler and more effective for surface meshing. It is extendable and can be easily applied. DelPhi [22], GRASP [23], MEAD [24], UHBD [25] and the PBEQ [26] module in CHARMM [27] are among the most common finite-difference-based PB solvers for computing biomolecular electrostatics, while the resolution of the molecular surface in the traditional finite difference method is restricted by the structure mesh size (Fig. 1). AFMPB [19] is a boundary element based PB solvers for biomolecular electrostatics. Tu et al. [15], presented finite-element-based PB and PNP solvers. FEMs and BEMs can deal with models of complicated geometries more effectively and accurately, especially when the geometrical shapes can be preferably approximated by the mesh [28].

Visual analysis is important in scientific computations. So far, there have been many tools for molecular visualization. The visual molecular tool can lead to biophysical development. For instance, the visualization of protein-ligand complex is critical in elaborating protein-ligand interactions and aiding novel drug design. Visualizing three-dimensional (3D) molecular structures of biopolymers has become a routine in the life sciences, e.g., ligand-binding pockets or other details in macromolecular assemblies help to elucidate the relationship between protein structure and function.

This paper aims to summarize the various definitions of molecular surfaces, review the state-of-the-art methods for molecular surface remeshing, including our recent work, and introduce their applications in implicit solvent modeling and simulation. In addition, applications of molecular remeshing and molecular visualization tools are also explored. The remainder of this paper is structured as follows. In “Molecular surface definitions and meshing tools” section, the various definitions of molecular surfaces, corresponding meshing methods, and remeshing methods are introduced. In “Mesh generation and surface remeshing” section, challenges in surface remeshing are displayed and some remeshing methods are introduced. In “Application of molecular surface mesh” section, applications of molecular surface meshing and our recent work regarding the membrane-channel protein system and volume mesh generation are described. “Molecular visualization systems” section summarizes the development of visual tools regarding desktop applications and online viewers, and “Conclusion” section presents the concluding remarks.

## Methods

### Molecular surface definitions and meshing tools

#### Definitions for molecular surface

The molecular surface is defined in various senses. The most widely used molecular surfaces include the van der Waals surface (VDWs) [29], the solvent accessible surface (SAS) [30], the solvent excluded surface (SES) [31], the minimal molecular surface (MMS) [32], the molecular skin surface [33] and the Gaussian surface [34].

The VDWs is defined as the surface of the union of the spherical atomic surfaces with the VDW radius of each atom within the molecule. Using the VDWs makes it convenient to calculate the molecular surface area and the surface normal, but it contains many pores that cannot contain water molecules.

The solvent accessible surface [30], also known as the Lee-Richards molecular surface, is the trace of the centers of probe spheres rolling over the VDWs. The radii of probe spheres are typically 1.4 Å. From the geometrical perspective, the SAS is equivalent to the VDWs obtained through increasing the VDW radius by the radius of a water molecule. The SES (also known as the “molecular surface” or “Connolly surface”) is the surface traced by the inward-facing surface of the probe sphere [31].

**Fig. 2:**
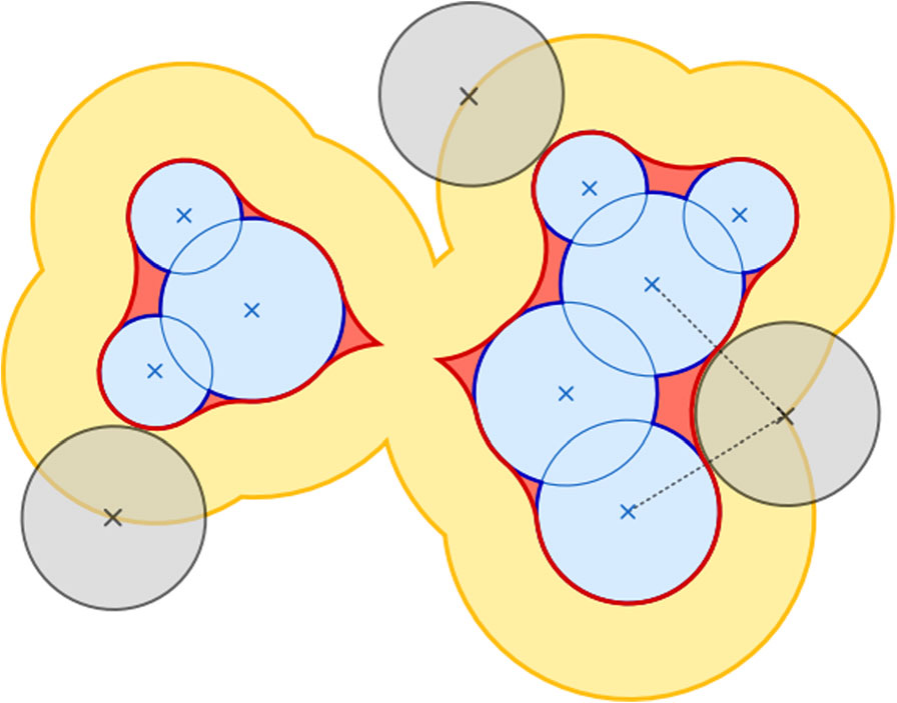
2D schematic of VDWs surface (blue), SAS (yellow), and SES (red). The SAS and SES are defined by a spherical probe (gray) that rolls over the VDW surface

In contrast to the VDWs, the SES contains less cracks and surface dimples. The SAS and SES are represented by the trajectory of the center and the inter-boundary of a rolling probe on the VDWs, respectively. Figure 2 shows an illustration of the VDWs, SAS and SES.

The skin surface is defined by a set of weighted points representing the atoms and a scalar called the shrink factor controlling the hyperboloidal connections between neighboring spheres. The skin surface is smoother than the VDWs and its tangent is continuous [33]. For the MMS, Wei et al. [32] constructed a surface-based energy function and used minimization and iso-surface extraction processes to obtain the so-called minimal molecular surface.

The MMS [32] is defined as the smallest area in all the surfaces of the VDW atoms containing proteins. Based on the theory of differential geometry, they proposed that the MMS can be determined by the mean curvature equation with constraints:

1 $$ \frac{dS}{dt}=\left\Vert \nabla S\right\Vert \nabla \cdot \left(\frac{\nabla S}{\left\Vert \nabla S\right\Vert}\right) $$

where *S*(*x*, *y*, *z*, *t*) is the hypersurface function.

Unlike the definitions above, the Gaussian surface [34] is defined as a level set of the summation of the Gaussian kernel functions as follows:

2 $$ \left\{\overrightarrow{x}\in {R}^3,\phi \left(\overrightarrow{x}\right)=c\right\} $$

where

3 $$ \phi \left(\overrightarrow{x}\right)=\sum \limits_{i=1}^N{e}^{-d\left({\left\Vert \overrightarrow{x}-{\overrightarrow{x}}_i\right\Vert}^2-{r}_i^2\right)} $$

The parameter *d* is positive and controls the decay speed of the kernel functions; *x*_*i*_and *r*_*i*_ are the location and radius of atom *i*, respectively; *c*and*d*are usually set as 1 and 0.5, respectively. Compared with other definitions of molecular surface, the Gaussian surface is smooth and more suitable to represent the electron density of a molecule [35]. These two parameters, *c* and *d* can be chosen such that the Gaussian surface approximates the SES, SAS and VDWs well [35, 36]. Compared with the other definitions, the Gaussian surface has the following advantages.

1. The Gaussian surface is smoother.
2. The Gaussian surface provides a realistic representation of the electron density of a molecule compared to other molecular surface definitions [35].
3. The Gaussian surface is well established [37–40] and has a wide range of applications in computational biology, such as docking problems [41], molecular shape comparisons [42], calculating SAS areas [43] and the generalized Born models [44].

#### Advances in biomolecular surface mesh generation tools

With the various definitions of molecular surface proposed, numerous works have been devoted to the computation of molecular surfaces. The biomolecular surface mesh substantiates that it is indeed a discrete representation of the biomolecular surfaces, which has a wide range of applications in the visualization, geometric calculation and solution of the implicit solvent model. The development of mathematical modeling and numerical simulation of biomolecular systems, especially the solution of implicit solvent model, proposes new requirements for the biomolecular surface mesh, such as high quality, efficiency and stability.

In recent years, as a variety of molecular surfaces definitions have been put forward, many kinds of algorithms for calculating the molecular surface meshes are constantly emerging, as described below. In 1983, Connolly [45, 46], proposed an algorithm for calculating the SAS and SES analytically. In the work, he separated the molecular surfaces into three parts: convex spherical surface, saddle-shaped toroidal surface and concave spherical triangular surface. These surfaces can be detected by the number of atoms touched by the probe. In 1995, a popular software, GRASP [23], for visualizing molecular surfaces was presented by Nicholls. In 1996, Sanner et al. [47] proposed an algorithm, called MSMS for generating triangular meshes based on an “reduced surface”, which is extremely useful for its high efficiency. MSMS contains four steps. First, it computes the reduced surface of the atoms. Second, it constructs the analytical representation of the SES based on the reduced surface produced in the first step. Third, the singularities created in the second step are handled. Finally, the SES is triangulated. MSMS is one of the most widely used software for molecular surface triangulation because of its high efficiency. In the next year, Vobrobjev et al. [48] introduced SIMS, a new method of calculating the solvent SES surface, which can eliminate the self-intersecting parts and the smooth singular regions of the SES. Ryu et al. [49] proposed a method based on Beta-shapes, which is a generalization of a shape [50]. In 2006, Can et al. [51] developed LSMS to generate the SES on grid points utilizing level-set algorithms. The software used the fast marching method to reach the molecular surface by propagating an initial seed surface.Yu et al. [39] designed a new tool GAMer, for mesh generation and quality improvement on the Gaussian surface. In 2008, Bajaj et al. [52] implemented a new program Molsurf to generate meshes on various types of molecular surfaces using high-order level-set methods. In 2009, Zhang et al. [53] presented a tool EDTsurf for mesh generation of the VDWs, SAS, and SES based on the LSMS algorithm. Chavent et al. [54] applied a ray-casting algorithm to visualize the molecular skin surface. In 2011, Chen et al. proposed a skin surface meshing software, known as TMSmesh [37, 55], for generating arbitrary macromolecules. In 2013, Decherchi et al. [56] presented NanoShaper, a software based on the ray-casting algorithm, that can generate surface meshes for the SES, molecular skin surface and Gaussian surface. NanoShaper primarily includes five parts [56]: 1) a surface build-up part, where the shape of the surface is calculated, analytically if possible; 2) a ray-casting part, where grid-consistent rays are cast, the corresponding intersections with the surface are collected, and the enclosed volume is estimated; 3) a cavity detection part, where the identified cavities are possibly removed according to their volume or shape; 4) a Marching Cubes part, where the surface is triangulated consistently with the previous cavity detection/removal and the corresponding surface area is calculated; and 5) a projection part, where a subset of the grid points are projected onto the surface, with steps 1), 2), and 4) for surfacing and steps 2) and 4) for triangular mesh generation. The surface is assumed to be a manifold. In the same year, Liao et al. [38] proposed a new mesh generation algorithm using multi core GPU and CPU accelerations.

In 2015, MSMS, Molsurf, NanoShaper, EDTsurf, TMSmesh and GAMer were compared and discussed by Liu et al. [34]. These methods or tools are typically successful in calculating the surface of small and medium biomolecules, most of which are not applicable to the calculation of large molecules. In addition, in the calculation of structural biology and structural bioinformatics, most methods are primarily used for the visualization of molecules, such as GRASP, MSMS, and LSMS, and the quality of mesh generation cannot reach the standard of numerical simulation. NanoShaper can quickly generate multiple surface meshes for biomolecules, but for molecules with complex shapes, the resulting meshes are not guaranteed to be manifold. TMSmesh [37] is a tool that can produce high quality meshes for biological macromolecules, but its operational efficiency requires further improvement. In this regard, Liu et al. proposed an improved version, i.e. TMSmesh 2.0 [55]. Their results show that TMSmesh 2.0 is robust, efficient and more than 30 times faster as compared to the previous version.

The reasons that TMSmesh 2.0 [57] is at least 30 times faster than the old version of TMSmesh are as follows: First, the new adaptive way of partition process to locate the surface reduces the number of surface-intersecting cubes and different sizes of cubes are used according to the approximation accuracy of the piecewise trilinear surface in the new method, instead of using the same-sized cubes in the previous method. A smaller number of cubes are used to precisely locate the surface.

**Fig. 3:**
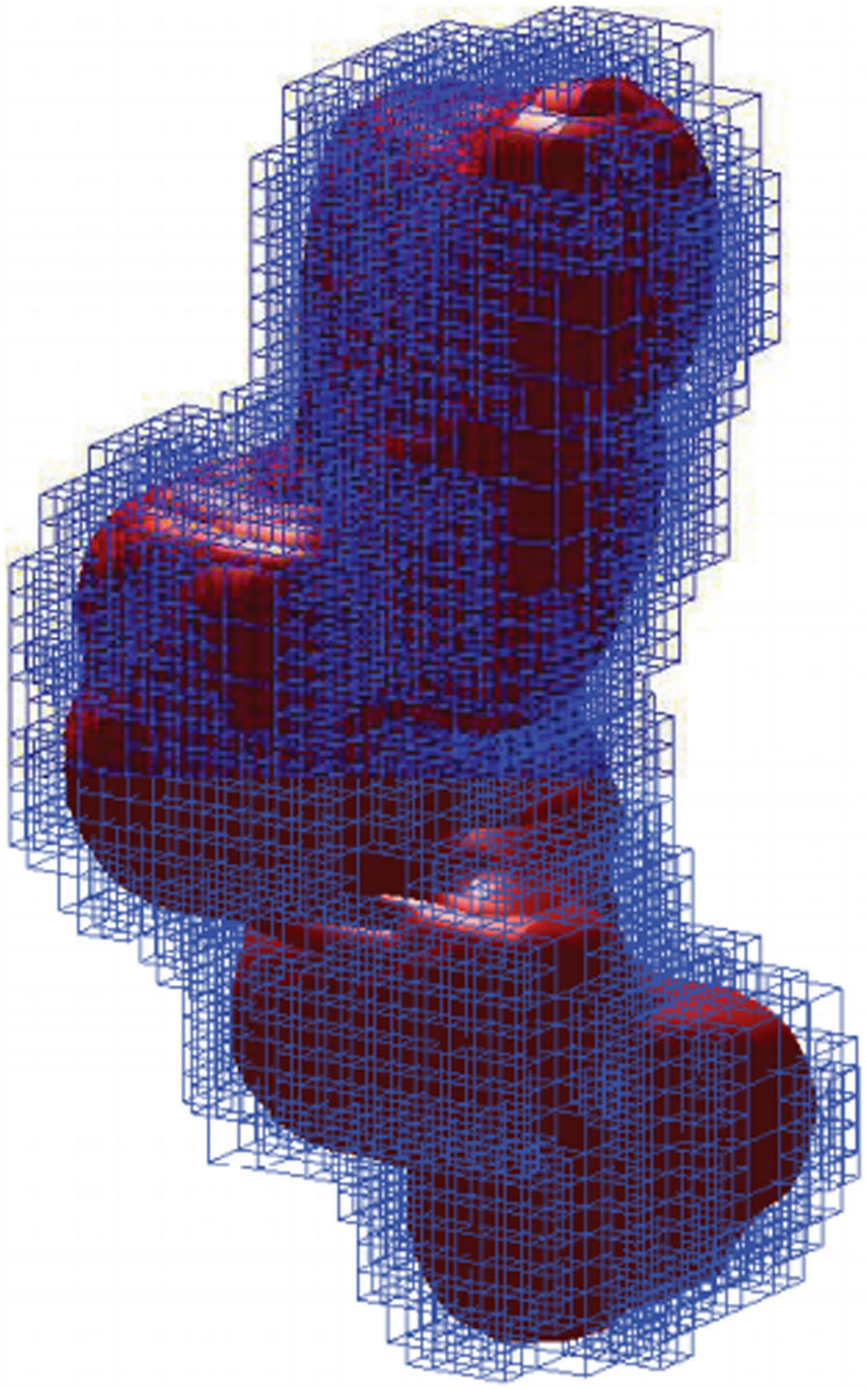
Cubes intersecting the Gaussian molecular surface for an ADP molecule. These cubes are represented in blue and the molecular surface is represented in red [57]

Second, a more efficient and much sharper bound estimator of the summation of Gaussian kernels in a cube is adopted as shown in Fig. 3.

**Fig. 4:**
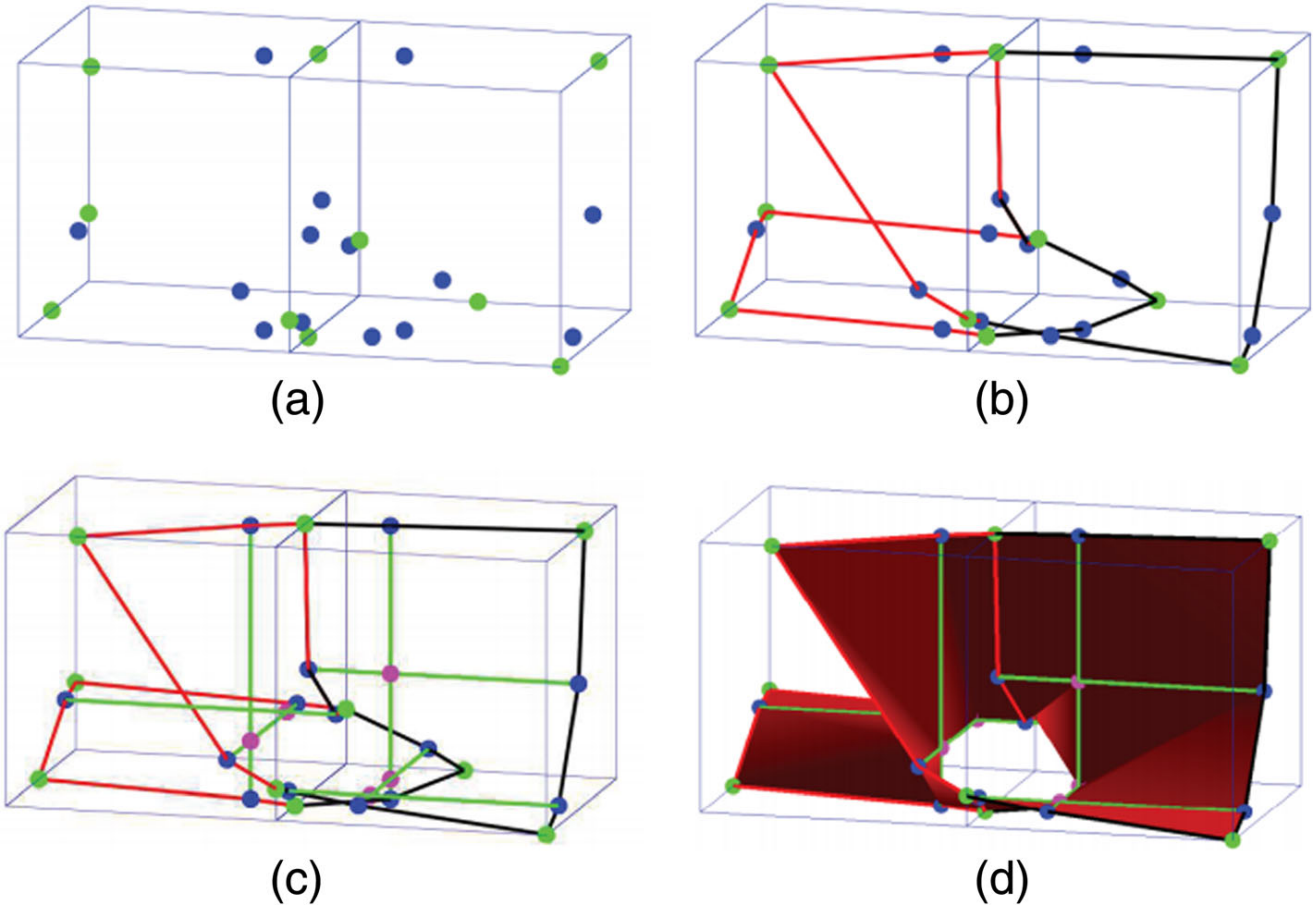
Method of triangulating the trilinear surface. **a** Step 1, Computing the intersection points and extreme points on the faces. **b** Step 2, Connecting intersection points and extreme points, forming a closed loop. **c** Step 3, Computing the fold curves and critical points. **d** Step 4, Dividing into single-valued pieces through the fold curves

Third, trilinear polynomials are used to approximate the surface, which reduces the computation cost significantly. For a trilinear surface (see Fig. 4), the surface points and fold curves can be computed explicitly, and the fold curves are explicitly straight lines, thus rendering the tracing process easier.

### Mesh generation and surface remeshing

#### Mesh generation

A mesh is a discretization of a geometric domain into small and simple units or elements [58], where triangles and quadrilaterals are most commonly used as the basic elements. This study is primarily concerned with triangular meshing. In addition to triangular meshing, quad meshing [59–62] and hexahedral meshing [63] are also important and involves further challenges due to their computational complexities. In this regard, a wave-based method [64] is used to remesh a given surface into anisotropic-sized quads. This method is capable of symmetric structure preservation and anisotropic/isotropic size control. Geometric objects are typically converted to meshes for efficient rendering and numerical solution of partial differential equations. Therefore, mesh generation becomes one of the essential steps for most geometry processing applications [65].

### Challenges in surface remeshing

Various approaches in surface remeshing have their own targeted goals and objectives. However, the following challenges are mostly considered for analysis of the applicability and validity of a specific approach [66, 67].

#### Geometric fidelity

The output mesh is typically required to have a best approximation of the input mesh geometry. Numerically, the approximation error is computed for analysis of geometric fidelity. Typically, the Hausdorff distance is used to estimate the approximation between the original input mesh and the improved one. A number of studies [68–70] have focused on the calculation of the Hausdorff distance. The Hausdorff distance can be one sided from the input mesh *M*_*i*_ to the output mesh *M*_*f*_, as calculated by Eq. (4), or two sided as calculated by Eq. (6) [66].

4 $$ {d}_h\left({M}_i,{M}_f\right)=\underset{p\in {M}_i}{\max}\left(p,{M}_f\right) $$

where*d*(*p*, *q*)is the Euclidean distance between two points *p* and *q* in a 3D space. The distance from point *p* to surface *M* is defined as the shortest distance between p and the nearest point in M, as given in Eq. (5)

5 $$ d\left(p,M\right)=\underset{q\in M}{\min }d\left(p,q\right) $$

Note that in one sided Hausdorff distance*d*_*h*_(*M*_*i*_, *M*_*f*_) ≠ *d*_*h*_(*M*_*f*_, *M*_*i*_). The two-sided Hausdorff distance is given in Eq. (6) [66].

6 $$ {d}_H\left({M}_i,{M}_f\right)=\max \left\{{d}_h\left({M}_i,{M}_f\right),{d}_h\left({M}_f,{M}_i\right)\right\} $$

#### Element quality

Quality improvement of mesh elements (edges, vertices, triangles) is an important goal in surface remeshing. Typically regular vertices are preferred. Short edges and triangles with small or large angles are avoided to improve the efficiency of numerical simulations [71]. Similarly, aspect ratio close to 0 is avoided [67, 72].

#### Validity and complexity

The output mesh should be valid. The validity of mesh ensures that the mesh is closed and a simple manifold [67]. The mesh complexity is typically computed as the number of elements. This number is usually required to be minimal, yet sufficient elements are required to ensure mesh quality and geometric fidelity [66].

#### Input mesh: uncertainties and defects

Prior to surface remeshing, the mesh is generated with any suitable method for a given application. In order to ensure a better approximation, the mesh is generated with high complexity, e.g., with 3D scanners [67]. Similarly, the meshes generated with TMSmesh [37, 55] for molecular surfaces also contain irregular elements, redundant vertices and complex geometries due to the irregular shapes of molecular surfaces. Therefore, surface remeshing becomes further challenging when the input mesh has several defects and complex structures.

### Meshing quality measurements

The main objective of quality meshing is to improve different quality parameters. The parameters used for meshing quality measurements in previous papers [55, 65, 66, 73] are described in the following. The triangle quality calculated for a triangle t is used for the mesh quality analysis which is given as

$$ Q(t)=\frac{6}{\sqrt{3}}\frac{A_t}{p_t{h}_t} $$

where *A*_*t*_ is the area of the triangle *t*, *p*_*t*_is its half-perimeter, and *h*_*t*_ is the length of its longest edge [74]. Typically, *Q*_min_(minimum quality) and *Q*_*avg*_(average quality) are used for analysis of the meshing results. Similarly, *θ*_min_(minimal angles) and *θ*_max_(maximal angles), are also used for comparison. In addition, ${\overline{\theta}}_{\mathrm{min}}$ representing the average value of the minimum angles in each triangle, and the percentage ratio of the small and large angles triangle are used. The area and volume preservation are also used for some of the applications [73, 75]. Similarly, for feature preservation, the Hausdorff distance is also used in the results analysis [66, 76]. The improvement in mesh regularity is also considered i.e. the ratio of the regular vertices. A regular vertex has a valance of six for the interior vertices and four for the boundary vertices. Furthermore, the aspect ratios (min, max) are computed using Eq. (7).

7 $$ AR=\frac{abc}{8\left(S-a\right)\left(S-b\right)\left(S-c\right)} $$

where *a*, *b*, and *c*are the lengths of the triangle’s edges and *S* = (*a* + *b* + *c*)/2 .

### Molecular surface remeshing

#### Molecular surface mesh generation pipeline

A benchmark for molecular structures in the PDB (protein data bank) and PQR formats can be found in the following website: (http://lsec.cc.ac.cn/~lubz/Meshing.html), which was used in the previous TMSmesh tests. In the PQR format, the occupancy and temperature factor columns of a PDB file is replaced with charge Q and radius R, respectively. These files are compatible with several popular computational biology tools [77]. The PQR files are used in TMSmesh [37, 57] for surface mesh generation. The surface mesh generated by TMSmesh 2.0 typically has a number of zero degree angles and redundant vertices, which requires further refinement. For example, SMOPT, ISO2mesh or Taubin method [78] can be used for mesh improvement at this stage.

#### State-of-the-art methods in molecular surface remeshing

In computer graphics, researchers have presented many surface remeshing methods. These methods include mesh simplification-based methods [79, 80], Delaunay insertion methods [81], advancing-front-based method [82], field-based approaches [83, 84], and local operators-based mesh optimization [85, 86]. In addition to these, global optimization methods are widely used, including parameterization-based methods [87, 88], discrete clustering [89], and direct 3D optimization [90–93]. Furthermore, segmentation-based meshing can use the input meshes as a segment prior to remeshing, which facilitates in sharp feature preservation [94, 95]. For implicit feature preservation, several efficient feature functions are proposed [66, 89]. Laplacian smoothing [96] is the simplest method that moves each vertex to the central position of its neighbor. Equation (8) computes the new position *v*_*f*_for a free vertex *v*_*i*_ as the median of the positions of the *n* vertices *q*_1_, *q*_2_, *q*_3_, ⋯, *q*_*n*_ in its one-ring neighborhood.

8 $$ {v}_f=\frac{1}{n}\sum \limits_{j=1}^n{q}_j $$

Taubin [78] presented a LowPass filter method by combining two Laplacian-like filters, one with a positive parameter and the other with a negative parameter. The method computes the new position *p*_*f*_ from the old position*p*_*i*_ using Eq. (9). Here, the weighting factor w is typically set to 1. Here, λ is the weighting factor, which is replaced by another weighting factor m = − (λ + e) with a small value e = 0.02. The parameter e is used to set the value of m to be just smaller than − λ. These two weighting factors, including l and m, are alternatively applied for the backward translation.

9 $$ {p}_f={p}_i+\lambda \sum \limits_{j=1}^n\omega \left({q}_j-{p}_i\right) $$

In the field of molecular modeling, Decherchi and Rocchia [97] triangulated complex manifold surfaces using the ray-casting method in the Nano-bioscience field. They provided an overview of the molecular surfaces in implicit solvent modeling and simulations utilizing the BEM and the FEM. TMSmesh [37, 55], (as described in “Background” section) is a software for generating arbitrary molecular surface meshes. The improved version TMSmesh 2.0 is being used for efficiently generating manifold surface meshes for biomolecules that exceed one million atoms with shape and feature preservations [34]. A new tool, named Molecular Finite Element Solver (mFES) [98], uses tetrahedral finite elements to calculate the electrostatic potentials of large molecular systems. ISO2mesh [99] is a free matlab/octave-based toolbox, which is widely applied for mesh generation and processing. In general, ISO2mesh is used to create tetrahedral meshes from surface meshes and 3D binary and gray-scale volumetric images, which include segmented MRI/CT scans. It is also used for molecular mesh smoothing; however, it cannot process self-intersecting triangle pairs and small angle triangles. Liu et al. [75] proposed an algorithm called SMOPT, which is used for molecular surface remeshing. They used local modifications on the mesh to improve the mesh quality, eliminate redundant vertices, avoid non-manifold errors and remove intersecting triangles. For mesh smoothing, SMOPT has improved the Laplacian smoothing that is given in Eq. (10).

10 $$ {p}_i=\left(1-\beta \right){q}_i+\frac{\beta }{N}\sum \limits_{j=1}^{N_i}{q}_j $$

where *β* ∈ (0, 1) is the parameter to control the rate of smoothing, *N*_*i*_represents the number of vertices in one ring and *q*_*j*_ represent the jth adjacent vertex in the one-ring of the ith vertex. The results of SMOPT show a significant improvement in the mesh quality. However, there still exist very small angles that destroy the quality of triangles. In our recent paper [73], we used a cut-and-fill strategy to remove invalid regions and to refill the holes. In addition, we used local operators to refine the local regions in the neighborhood of the small triangles. This method showed a significant improvement in minimal/maximal angle improvements, aspect ratios, and other meshing quality parameters. However, further improvements such as none-obtuse remeshing and the improvement in the adaptive density are possible in this method.

## Results and discussion

### Application of molecular surface mesh

#### Application to boundary element simulations of electrostatics

**Fig. 5:**
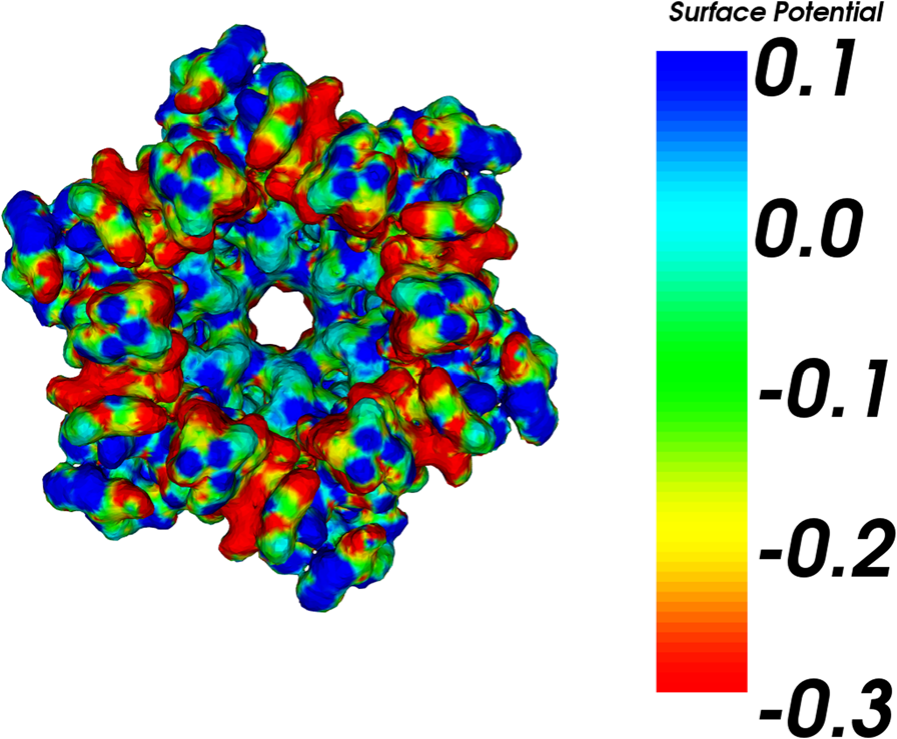
Electrostatic potential surface of Connexin calculated by AFMPB

We tested the meshes in boundary element calculations of the Poisson-Boltzmann electrostatics. The BEM software used is a publicly available PB solver, i.e., AFMPB. As a representative molecular system, we chose the structure Connexin. The initial coordinates for Connexin are obtained from the PDB (code GJB2 [100]). The surface mesh is generated by TMSmesh 2.0 and contains 101,574 nodes. Figure 5 shows the computed electrostatic potentials mapped on the molecular surface. The surface potential correctly captures the molecular charge property, which verifies the effectiveness and applicability of the mesh generated by our method.

#### Membrane-channel protein system mesh construction for finite element simulations

Over the past decades, little research has been conducted on the volume mesh generation of molecules in biophysics. In 2014, Tu et al. [16] built a tool chain to generate high-quality meshes for practical protein systems by combining a few mesh generation tools. The tool chain mainly consists of four components: surface meshing, quality improvement, volume mesh generation and membrane mesh construction.

The triangulated molecular surface can be generated by some surface mesh generators as shown in “Advances in biomolecular surface mesh generation tools” section. If necessary, the surface mesh quality can be improved by software such as ISO2mesh [99] and TransforMesh [101]. Once the surface mesh is generated, and the mesh has no manifold errors, the tetrahedral volume mesh of the system, which consists of the molecule and the solvent box, can be generated by the TetGen software [102].

**Fig. 6:**
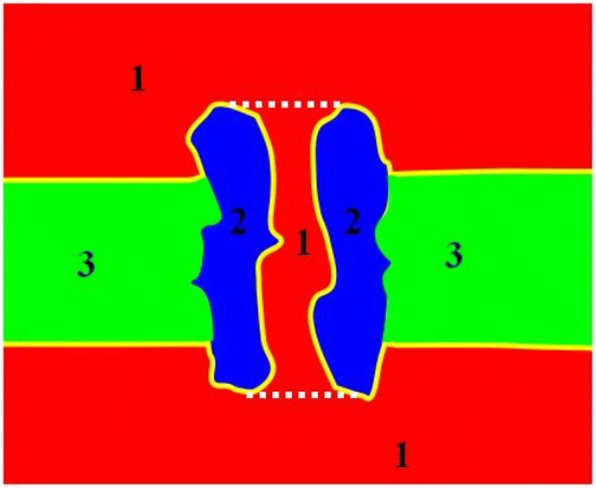
Schematic 2D picture for the cross section of an ion channel system. The solvent region is labeled 1, the channel protein is labeled 2 and the membrane is labeled 3. The solvent part between the dotted white lines is the pore region [79]

**Fig. 7:**
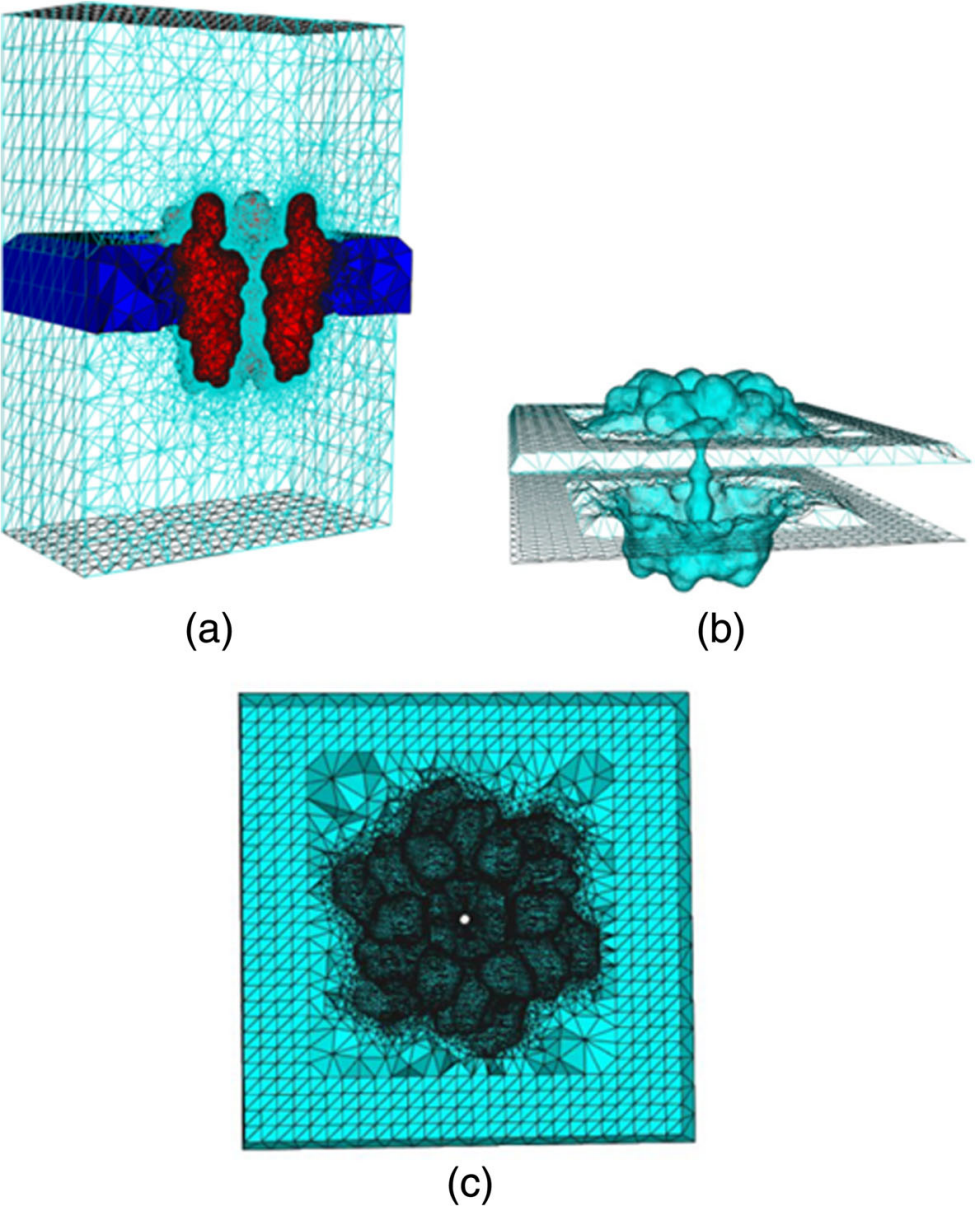
Volume mesh of Connexin: **a** Wire-frame of volume mesh conforming to the boundary of a channel protein and membrane system, **b** the surface mesh of the membrane-protein region, **c** the upper boundary surface of the membrane-protein region, in which the membrane is represented as a slab [89]

For transmembrane proteins, the mesh needs to add membrane information, as shown in Fig. 6. Therefore, the final step is to generate the membrane volume mesh. This tool chain is suitable for arbitrary-sized molecules, but for transmembrane proteins, especially membrane-channel protein system, membrane volume mesh generation is a hard problem, because the inner shape of the ion channel is highly complex. The key point of Tu’s method in membrane construction is marking the tetrahedra belonging to the membrane region correctly and obtaining the triangles on the surface of the membrane-protein region. They use one or more manufactured spheres or cylinders to separate the membrane and pore regions. However, their method requires to locate the spheres or cylinders manually depending on the structure of a given ion channel. A universal sphere that is suitable for different ion channels is not available. In 2015, Liu et al. [103] presented an algorithm to detect the inner triangulated surface of the pore to separate the membrane region and the pore region in tetrahedral mesh (Fig. 7).

Currently, parallel computers enable us to solve a problem with a mesh containing tens of millions of vertices. However, CPU time and memory limitations still challenge the generation of a large high-quality mesh on a single machine. Obviously, a parallel environment significantly reduces the amount of time required for large-scale mesh generation.

In 2016, Xie et al. [104] proposed a method to generate tetrahedral meshes in parallel, directly from the initial molecules. Their code was based on the parallel adaptive finite element package PHG [105], which was used as a message-passing interface. PHG provides many interfaces for computation and meshing and hides the parallelization details for an easy implementation with thousands of CPU cores. Using the adaptive mesh refinement interfaces in PHG, they successfully developed and implemented parallel unstructured mesh generation algorithms for a given molecular PQR file.

**Fig. 8:**
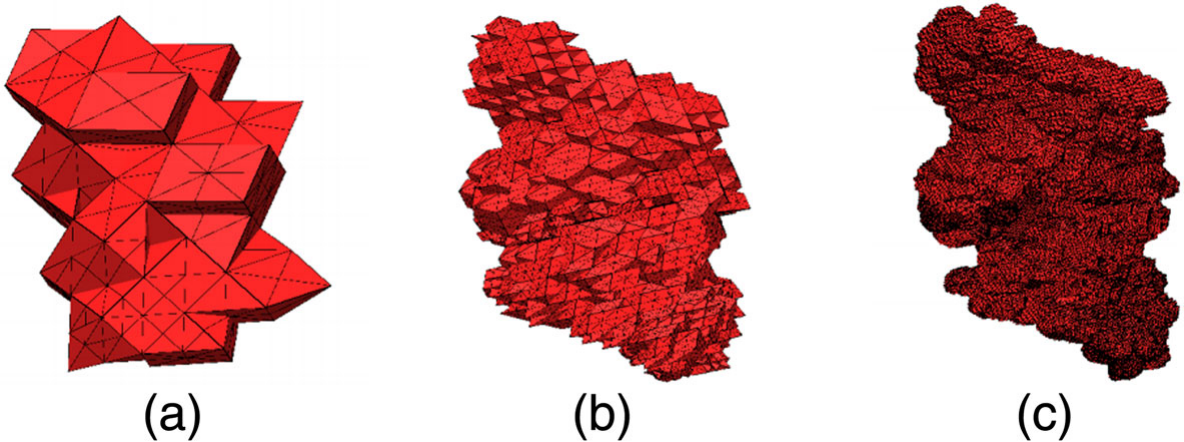
Solute surfaces of the DNA fragment after a number of refinements: **a** 6, **b** 12, and **c** 18 [90]

The meshes in Fig. 8 begin from a background mesh that contains 129 vertices and 342 tetrahedra and are obtained after completing 6, 12 and 18 mesh refinements separately after eight uniform refinements using Xie’s method (Fig. 8).

### Molecular visualization systems

#### Desktop applications

**Fig. 9:**
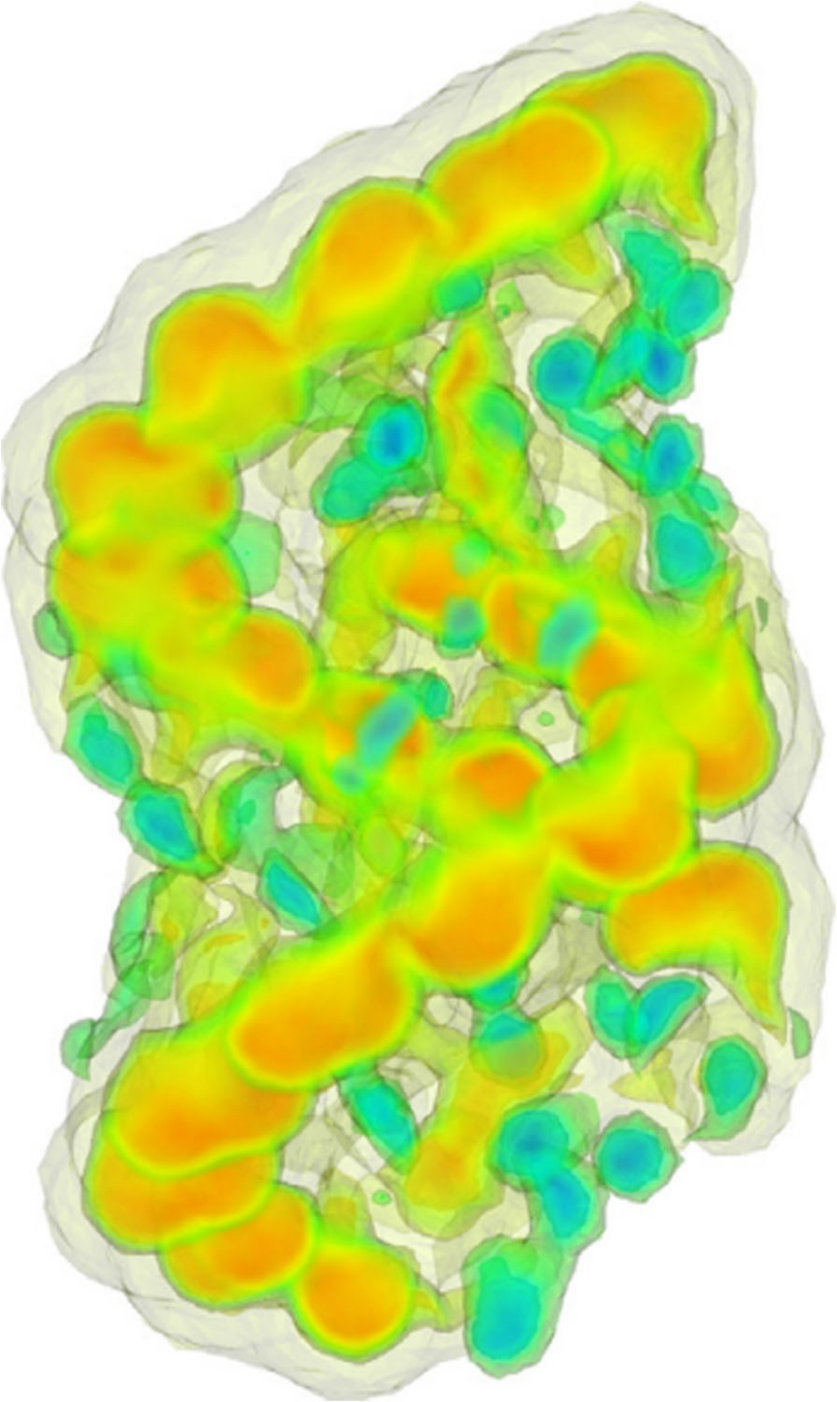
Volume rendering of electrostatic potential around a DNA fragment via VCMM [94]

To date, dozens of visualization tools are available for molecular visualization. PyMOL (http://www.pymol.org), VMD [106] and Chimera [107] are among the most popular ones currently. They can interpret multiple file formats and generate multiple representations to supply precise and powerful control. However, these software packages lack the capability of unstructured mesh management and visual analysis of numerical results based on unstructured meshes. Furthermore, these packages do not provide functions for BEM and FEM simulations. Therefore, a molecular visualization tool with these functions is urgently required. VCMM [108], is used for continuum molecular modeling with focal applications of the BEM and FEM. It can manage the unstructured mesh and the macromolecule mesh. This software was designed for the numerical results of BEM and FEM solvers (Fig. 9).

#### Web application

Due to the requirement nature of three-dimensional (3D) graphics, most molecular viewers are desktop applications. The need to install specialized applications, and occasionally, the restriction of software licenses, results in hurdles to the sharing of molecular data. Unlike a desktop application, a standards-based client-side web application comes pre-installed with every computer and mobile device with a modern web browser and can be seamlessly integrated into online environments for accessing and analyzing molecular data. Subsequently, web browsers need to display 3D content with additional plug-ins. Next we will discuss a few applications for molecular visualization.

Jmol [109] is implemented as a Java applet and includes a custom rendering engine for efficiently rendering common molecular data representations, such as spheres and sticks. Because of this custom rendering engine and Java’s optimizing just-in-time compiler, the performance of Jmol can approach that of desktop applications mentioned above. However, as a result of heavily publicized security failures, the Java install base is shrinking. Even when the Java is installed, users are presented with multiple security prompts that must be correctly navigated before a Java applet can run. Therefore, programs such as Jmol are needed to be embedded as Java Applets within a web page, for instance, OpenAstexViewer [110], or web visualizer base on Jmol, such as GIANT [111], both of which are so convenient. Similarly, JSmol [112] is another product of applying Java or JavaScript translator to Jmol. For large and complex visualizations, however, the performance of JSmol lags behind that of Jmol.

**Fig. 10:**
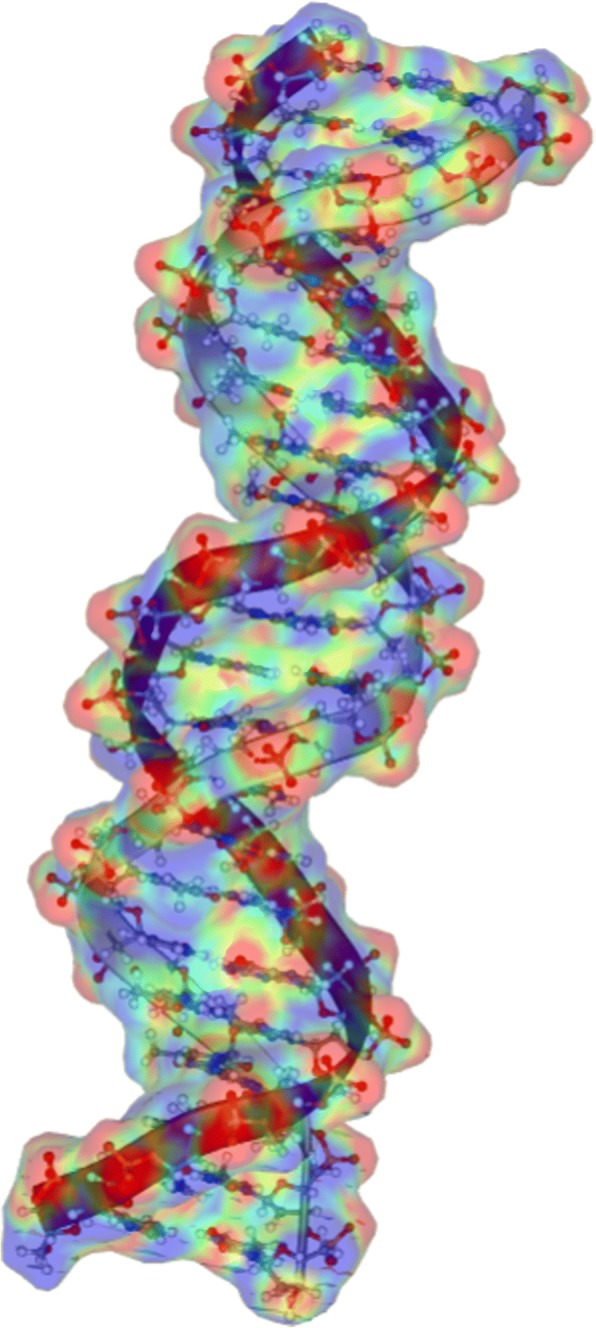
The DNA ball-stick model, secondly structure and electrostatic potential surface by VISM

In contrast, WebGL visualizers such as ChemDoodle Web Components (http://web.chemdoodle.com) benefit from GPU acceleration. PV [113] and 3Dmol.js [114] are two examples of WebGL-based molecular viewers. They provide an API for the creation of molecular visualizations. As the first WebGL viewer, GLmol [115] uses the Three.js(http://threejs.org) framework for interfacing with WebGL. It features an experimental version of surface construction based on the EDTSurf algorithm. However, GLmol lacks a full featured API and the use of the Three.js library leads to performance inefficiencies as well. In 2015, Alexander et al. [116] proposed a new viewer, NGL Viewer, which provides a richer graphical user interface (GUI) for customization of molecular scenes in addition to a developer API for embedding and controlling the viewer. Leveraging the features of modern web browsers, the NGL Viewer that can supply fast is based on GPU to hardware-accelerated molecular graphics and brings a familiar GUI to the web. It offers a general molecular visualization function which does not require the installation of specialized software and can simplify the access of 3D structural data for biology scientists. The NGL viewer can be embedded into other websites by including a single JavaScript first and then calling the API methods to create a scene instance that allows loading and subsequent manipulation of molecular structures. The VISM (Fig. 10), as a product embedding the NGL’s API, can be used from webpage (www.xyzgate.com).

## Conclusion

We started with the definitions of various molecular surfaces including the van der Waals surface (VDWs), the solvent accessible surface (SAS), the solvent excluded surface (SES), the minimal molecular surface (MMS), the molecular skin surface and the Gaussian surface.

Then, we presented a comprehensive review of the molecular surface remeshing techniques. We also presented the applications of molecular surface remeshing. In addition, our recent work about membrane-channel protein system and volume mesh generation were also summarized. Furthermore, few commonly used tools for surface remeshing and molecular visualization have also been discussed.

There are few directions of further work in molecular surface remeshing and related research. The surface remeshing methods are dependent on the input meshes, as described in “Mesh generation and surface remeshing” section. However the mesh generation from PQR files, which is typically achieved with TMSmesh [37, 57], gives complex and raw type meshes.

**Fig. 11:**
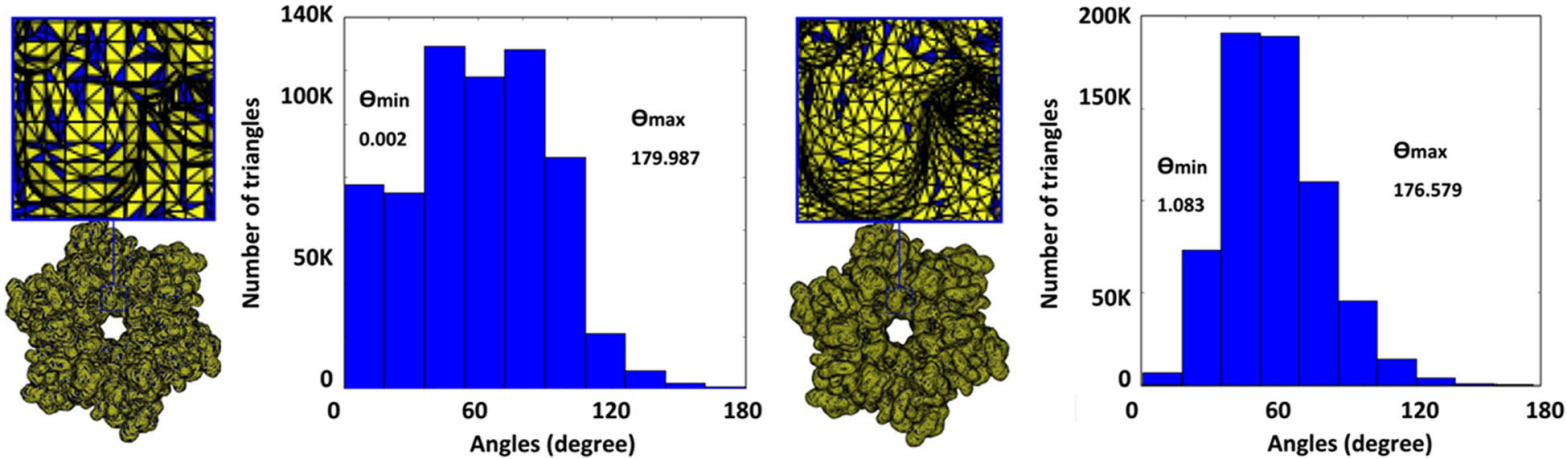
Example of molecular surface mesh (Molecular name/PDB ID: Connexin). The blue color indicates triangles with angle < 30. Left: Mesh generated by TMSmesh 2.0 from PQR file (with 107,500 vertices, Ө min = 0.002, Ө max = 179.99, angle < 30° = 56.21%, and regular vertices = 75.87%). Right: Mesh improved with SMOPT (with 105,645 vertices, Ө min = 1.08, Ө max = 176.58, angle < 30° = 16.98% and regular vertices = 76.78%)

The mesh generated with TMSmesh has a number of small angles and short edges which are difficult for further improvement (see Fig. 11 (left)). Therefore, a more robust algorithm with an easy-to-use interface is demanded to generate a good quality mesh from the PQR files. Similarly, for the improvement of the generated mesh, only few works are available. For example, the mesh improved with SMOPT (see Fig. 11(right)) still have a high ratio of small angles and further improvement is strongly required. Though our recent work [73] has improved the minimal angles and some other quality measurement, the maximal angle and the adaptive remeshing are still important for consideration. Furthermore, for volume mesh generation and simulation, new tools and methods can be introduced. Molecular surface remeshing and molecular visualization is a cross-disciplinary field that overlaps with several fields including molecular biology, bio-physics, mathematical modeling, and computer graphics. Therefore, easy-to-use software products for the end-users are also demanded in the field.

## Abbreviations

PB: Poisson–Boltzmann
PDB: protein data bank
PNP: Possion- Poisson-Nernst-Planck
AFMPB: adaptive fast multipole Poisson–Boltzmann

## About the authors

Sheng Gui is currently a master candidate with the Institute of Computational Mathematics and Scientific/Engineering Computing, Chinese Academy of Sciences. His research interests include biomolecular mesh generation and molecular visualization.

Dawar Khan is currently a PhD candidate at the National Laboratory of Pattern Recognition of the Institute of Automation, Chinese Academy of Sciences. His research interests include computer graphics, surface remeshing and visualization.

Qin Wang is currently a PhD candidate with the Institute of Computational Mathematics and Scientific/Engineering Computing, Chinese Academy of Sciences. Her research interests include parallel finite element method in computational biology.

Dong-Ming Yan is currently an associate professor at the National Laboratory of Pattern Recognition of the Institute of Automation, Chinese Academy of Sciences. His research interests include computer graphics, geometric processing and visualization.

Ben-Zhuo Lu is currently a professor with the Academy of Mathematics and Systems Science, Chinese Academy of Sciences. His research interests include numerical methods, and application software.
